# Acute Effects of Cannabis Concentrate on Motor Control and Speed: Smartphone-Based Mobile Assessment

**DOI:** 10.3389/fpsyt.2020.623672

**Published:** 2021-01-22

**Authors:** Leah N. Hitchcock, Brian L. Tracy, Angela D. Bryan, Kent E. Hutchison, L. Cinnamon Bidwell

**Affiliations:** ^1^Institute of Cognitive Science, University of Colorado-Boulder, Boulder, CO, United States; ^2^Department of Health and Exercise Science, Colorado State University, Fort Collins, CO, United States; ^3^Department of Psychology and Neuroscience, University of Colorado-Boulder, Boulder, CO, United States

**Keywords:** cannabis (marijuana), dab, tapping, acceleration, speed

## Abstract

**Background:** The use of cannabis concentrate is dramatically rising and sparking major safety concerns. Cannabis concentrate contains tetrahydrocannabinol (THC) potencies up to 90%, yet there has been little research on motor impairment after concentrate use (commonly referred to as “dabbing”). This study measured postural control and motor speed after the use of high potency concentrates in males and females.

**Methods:** Experienced concentrate users (*N* = 65, Female: 46%, 17 ± 11 days/month of concentrate use) were assessed for motor performance in a mobile laboratory before, immediately after, and 1 h after *ad-libitum* cannabis concentrate use. Plasma levels of THC were obtained via venipuncture at each timepoint. We used a remotely deployable motor performance battery to assess arm and leg movement speed, index finger tapping rate, and balance. The sensors on a smart device (iPod Touch) attached to the participant provided quantitative measures of movement.

**Results:** Arm speed slowed immediately after concentrate use and remained impaired after 1 h (*p* < 0.001), leg speed slowed 1 h after use (*p* = 0.033), and balance decreased immediately after concentrate use (eyes open: *p* = 0.017, eyes closed: *p* = 0.013) but not at 1 h post-use. These effects were not different between sexes and there was no effect of concentrate use on finger tapping speed. Acute changes in THC plasma levels after use of concentrates were minimally correlated with acute changes in balance performance.

**Conclusions:** Use of cannabis concentrates in frequent users impairs movement speed and balance similarly in men and women. The motor impairment is largely uncorrelated with the change in THC plasma levels. These results warrant further refinement of cannabis impairment testing and encourage caution related to use of cannabis concentrates in work and driving settings.

## Introduction

The use of concentrated forms of cannabis, often referred to as “dabbing,” has become increasingly popular (1–4). Advances in production technology have allowed wax or resin dabs (5–7) to contain much greater concentrations of cannabinoids than more typical flower cannabis products. These concentrates often contain high levels of tetrahydrocannabinol (THC), the main cannabinoid associated with psychoactive effects from cannabis. Concentrates, with up to 70–90% THC potencies, are perceived by heavy concentrate users to be more dangerous than flower products, now averaging 10–30% THC (7–11), increase blood levels of THC (12), are associated with illicit drug use (1), higher rates of cannabis use disorder (2) and decreased mental and physical wellness (4, 13). However, the only report of acute physical effects of high-potency cannabis concentrate use that we know of is with a sample of flower and concentrate users in our prior publication (12).

The last two decades of research demonstrate that low-potency cannabis [i.e., up to 7% THC; (14) or 12 ng/ml plasma THC (15)] can impair executive function (16) as well as complex psychomotor performance. This includes maintenance of driving speed, reaction time, joystick errors (17), and simulator driving ability (15, 18–20). Complex psychomotor tasks like these can be sensitive enough to detect acute cannabis intoxication in chronic users (16). For example, low-potency THC was shown to acutely impair visuomotor arm tracking (in participants with a range of histories) (17). Low-potency cannabis effects have also been observed to be dose-dependent (21, 22), which has contributed to the rationale for current legal limits for THC whole blood or plasma levels of 5 or 7–10 ng/ml, respectively (23, 24). For instance, low-potency cannabis use modestly increased the risk of accident involvement in a driving simulator, but this was highly dose- and task-dependent (15). Complex psychomotor impairments from cannabis can therefore be observed in frequent users but are often dependent on dose and task complexity.

Psychomotor tasks that require high cognitive loads and controlled settings (i.e., driving simulations) often lack the precision to detect basic motor impairment [i.e., without enhanced intoxication from combining drug use (25–27)] and so far lack the external validity for use after naturalistic administration of concentrates (containing such high THC potencies). Greater understanding of driving capability after concentrate intoxication requires assessment of basic motor performance, such as the rapid movements necessary for safe driving behavior. In past research, administration of low-potency THC in cannabis users (≥30 total uses) produced subjective intoxication and decreased a common measure of basic motor performance (finger tapping speed), but was uncorrelated with THC plasma levels (28). Similarly, we recently demonstrated that unperturbed balance is acutely impaired after naturalistic use of higher potency cannabis (12). These findings suggest that concentrates may impair other basic motor tasks necessary for successful driving.

To better understand the effects of concentrated cannabis on basic motor performance, potential sex differences should be considered. With few exceptions, sex differences have been poorly characterized in frequent or heavy cannabis users (16), even though men typically consume cannabis more often and in greater quantities than women (29, 30). Medical marijuana laws have led to decreases in automotive fatalities for both men and women, but decriminalization of cannabis led to increases in fatal crashes for men only (31). After legalization, the changing patterns of use and the greater THC plasma levels that arise from concentrate use suggests the need for more detailed information on the basic motor effects after acute intoxication from concentrates (21, 26, 30–33). Low-potency THC administration decreased tapping speed of the non-dominant hand in women more than men (34), yet dominant-hand speed, especially after concentrate use, remains untested between sexes. Another measure of basic motor performance, balance, is similar between healthy men and women in most conditions (35, 36), yet the potential sex effect after cannabis use has not been investigated. Additionally, low-potency cannabis has been shown to decrease complex psychomotor speed more for men than women (37), but the effect of high-potency cannabis on basic motor performance alone has not been assessed.

Using a portable, smart-device based protocol in a mobile laboratory, we previously documented acute cannabis-induced balance impairment in a large sample of flower and concentrate users (12). Here, we examined the use of cannabis concentrate on our complete portable battery of motor tasks in only the concentrate user sample from our previous study (12). Measures were taken before, immediately after, and 1 h after use. The presentation of the balance data here, as compared with our previous paper, allowed us to examine: (1) sex differences in motor impairment, (2) repeated testing effects by trial, (3) correlations between THC plasma levels and motor performance, and (4) inter-task correlations for the entire battery of motor measures: balance under three different conditions and speed of arm extension, leg withdrawal, and finger tapping.

## Methods

### Participants

Methodological details pertaining to this sample population, baseline surveys, mobile lab procedures, cannabis potency, cannabinoid analysis, and the balance task are previously published (12) and are summarized below. Participants were recruited from the Boulder-Denver area in Colorado using social media and mailed flyers that summarized study criteria. Trained research staff screened potential participants via telephone. Study participants were oriented to the procedures and provided written informed consent. All procedures were approved by the University of Colorado-Boulder Institutional Review Board in accordance with the standards of the relevant national and institutional committees on human experimentation and with the Helsinki Declaration of 1975 as revised in 2008.

Criteria for enrollment included: (1) aged 21–70, (2) cannabis concentrate use ≥4 times in the past month and general cannabis use ≥1 year, (3) experience with 90% THC (highest potency cannabis that could be assigned for the study), (4) no non-prescription drug use in the past 60 days, except cannabis, (5) no daily tobacco use, (6) drinking ≤ 2 times per week with ≤ 3 (women) or 4 (men) drinks per occasion, (7) no pregnancy or intention to become pregnant, and (8) no current or history of psychosis or bipolar disorder. The age criteria (range: 21–69 years) were formulated to include a wide range of healthy cannabis users in the community in order to provide generalizable data on motor effects after concentrate use across various age groups.

A total of 75 concentrate users consented to undergo phlebotomy for plasma cannabinoid levels and smartphone-based testing of motor performance in the mobile laboratory vehicle at Pre-Use, Acute Post-Use, and 1 h Post-Use timepoints. Participants that did not complete key motor outcomes and/or did not have plasma data collected that conformed to our criteria (i.e., THC threshold of ≥20 ng/ml at the Acute Post-Use timepoint) were omitted from analysis (*n* = 10). Therefore, the sample of concentrate users studied for this report (*N* = 65) is nearly identical to a previous report of ours [*N* = 66 (12)], however, seven subjects differ between the study samples. Three participants did not complete key neurobehavioral outcomes and were omitted in our previous report. However, those three completed key motor outcomes and were therefore included in this report. Similarly, four participants completed key neurobehavioral outcomes and were included in our previous report, however, those four did not complete key motor outcomes and were omitted from this report.

### Study Visits

#### Baseline Session (Campus Visit)

**Figure 1:**
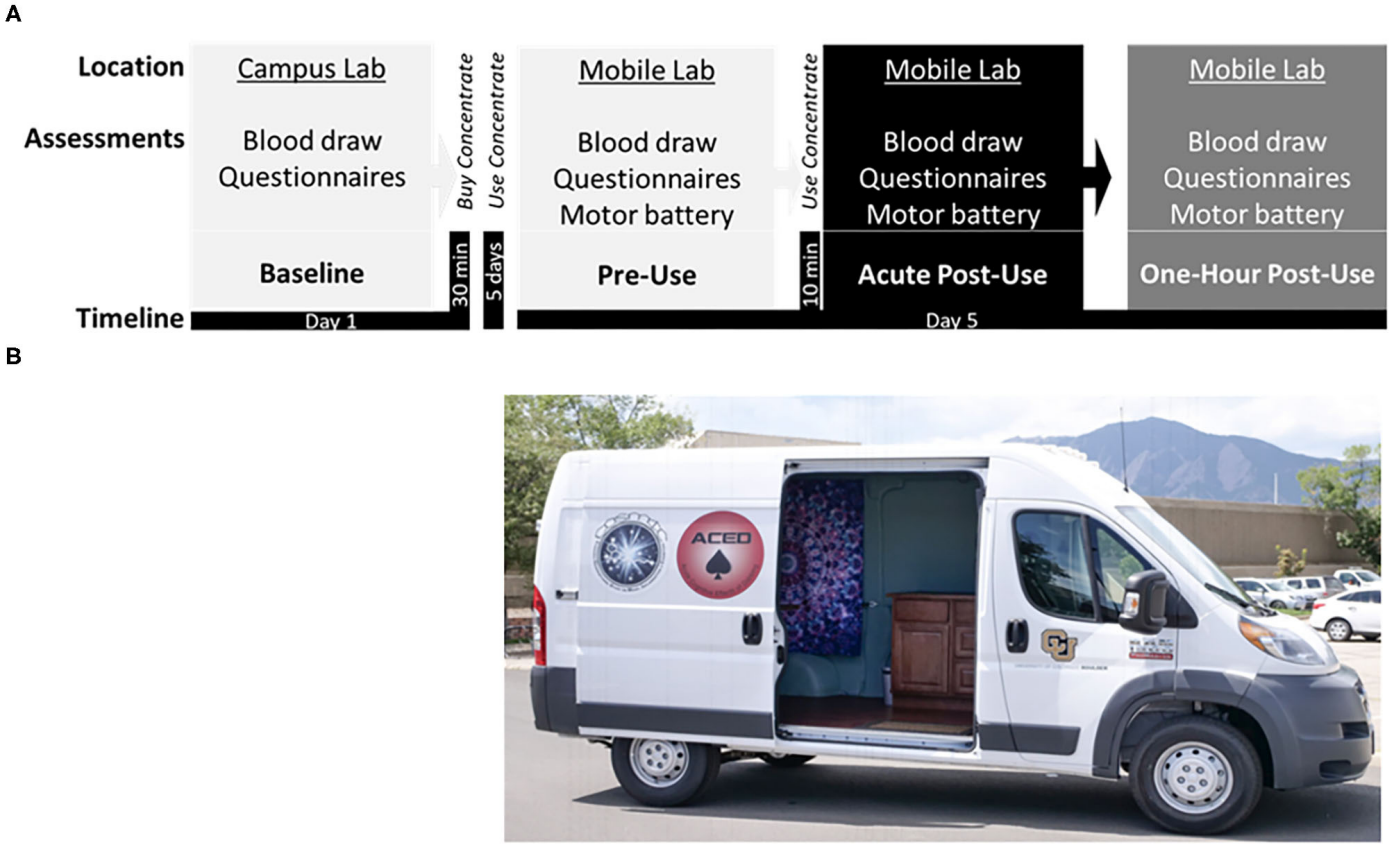
**(A)** Study timeline displaying the: Baseline session at the campus lab, followed by purchase of cannabis concentrate (70 or 90% THC) at a local dispensary, ~5 days of *ad libitum* use, and the subsequent Experimental session in the mobile lab, including the pre-concentrate use (Pre-Use) timepoint followed by in-home participant *ad libitum* use of concentrate, and two post-concentrate use timepoints (Acute Post-Use and 1 h Post-Use). **(B)** Photograph of the Mobile Lab, a high-top cargo van retrofitted with a Wi-Fi hotspot, hand rail and stair step, ice cooler, electrical outlets, a reclining phlebotomy chair, sterile equipment, and a chair/table for motor testing.

The Campus appointment included a 1.5-h visit (Figure 1). Participants were asked to refrain from alcohol or other recreational drug use for 24 h, cannabis use the day of testing, and tobacco or caffeine products for 1 h prior to the baseline appointment. Upon arrival, participants reviewed and completed the informed consent, a breathalyzer assay (Alcosensor IV, Intoximeter, Inc.; St. Louis, MO), a urine toxicology screen (SafeCup III Clia Waived, Germaine Laboratories; San Antonio, TX), and (for female participants) a pregnancy test (Sure-vue, Fisher Healthcare; Tulane, CA) to ensure that recent drug use or pregnancy were not present. Participants completed a blood draw, neurocognitive tests, and questionnaires.

At the appointment, participants were assigned to a concentrate potency condition (based on a random number table generated by the study statistician) and asked to purchase the assigned product at a local dispensary (The Farm; https://thefarmco.com/). Two concentrate products (70 or 90% THC potency) were set aside for participants to purchase. Federal regulations require that researchers not handle or blind the legal market products for participants. Differences between the two concentrate potencies (70 vs. 90%) were not observed with prior biological or psychomotor outcomes (12) and thus were not directly tested in current data analysis.

#### Experimental Session (Mobile Visit)

After the baseline appointment there was a 5-day *ad libitum* period for subjects to become familiar with the cannabis concentrate product. After this period, the second and final visit took place in a mobile laboratory (Figure 1). Before the mobile laboratory visit participants were asked to refrain from using alcohol or other recreational drugs for 24 h, cannabis use the day of testing, and tobacco or caffeine products for 1 h in preparation for three blood draws over 3-h. The mobile laboratory setting necessitated the use of portable technology to assess self-report surveys, plasma cannabinoid levels, and motor performance.

The experimental session (mobile visit) included three testing timepoints: before (Pre-Use), immediately after (Acute Post-Use), and 1 h after (1 h Post-Use) cannabis concentrate use. Assessments at each timepoint were performed identically and involved a blood draw, neurocognitive tests, questionnaires, and the motor battery. Participants completed the Pre-Use assessments, returned to their residence to weigh and use their desired amount of concentrate product, and were asked to immediately return to the mobile lab for Acute Post-Use and 1 h Post-Use testing.

### Demographics and Cannabis Use Questionnaires

During the baseline visit, participants reported their age, sex, race, height and weight for body mass index (calculated), and age of regular cannabis use onset via questionnaire. The Marijuana Dependence Scale [MDS (38)] measured dependency symptoms. The calendar-assisted Timeline Follow Back [TLFB (39)] interview queried participants drug use over the past 30 days. During the experimental mobile laboratory session, the mode of administration [i.e., glass dab rig/tube used primarily (12)], the amount of time participants administered concentrate (in their home), and the amount of concentrate participants reported using was recorded.

### Plasma Cannabinoids

A certified phlebotomist collected 32 mL of blood at each timepoint through venipuncture of a peripheral arm vein using standard, sterile phlebotomy techniques to assess plasma cannabinoid levels. Plasma was separated from erythrocytes, stored at −80°C, and sent to the Department of Anesthesiology at the University of Colorado Denver. Two plasma cannabinoids were quantified, THC and 11-OH-THC [the active metabolite with pharmacological activity (40)] using validated high-performance liquid chromatography/mass-spectrometry (41). Less than 5% of all cannabinoid values (22/450 data points) were below the quantifiable limit (<0.32 ng/ml), therefore 0.00 was replaced for those absolute values. Notably, no values less than that lower limit of quantification were observed at the Acute Post-Use timepoint. To ensure that participants followed study instructions and should be included in this analysis, the following cannabinoid criteria were set: (1) a THC measurement was obtained at Acute Post-Use, (2) THC value ≥20 ng/ml at Acute Post-Use, and (3) THC must have increased from Pre-Use levels.

### Motor Battery

#### Materials, Setup and Data Processing

A smart device (iPod Touch 5th generation, iOS 12.11, Apple Inc., CA) and data logging App (Sensor Data, Wavefront Labs) recorded the outcomes for the motor battery tasks. Research assistants described and demonstrated each task briefly and provided reminders of technique between tasks. Each task was completed twice, with a rest period of 30s between trials. Data were transferred to a lab computer and imported into the Spike 2 software program (Spike 2, v. 7.14, Cambridge Electronic Design, Cambridge, UK) for visual inspection and analysis. *Motor Battery* Supplementary Material provide materials, setup, and processing details for all tasks.

#### Tasks

##### Arm Extension

The goal of this task was to assess the ability to use a ballistic contraction to rapidly accelerate the arm over a small distance as is sometimes required during driving. The task was a standardized, abbreviated horizontal punch movement (a “jab”).

Setup: An iPod was firmly attached to the distal side of the participants forearm (above the wrist) with the arm at a right-hand angle, while in a seated position.

Directions: Participants were instructed as follows: “*Every time you feel a tap on the iPod, punch your arm straight out as fast as possible and bring your arm back to the starting position*”. Ten trials of the rapid arm movement were performed. A pseudo-random, investigator chosen, inter-trial interval of 2–5 s was employed to minimize the ability of the participant to predict the next tap stimulus and reduce confounding anticipatory movements. During this task participants kept their eyes closed, feet on the ground, and non-dominant hand in their lap. Two trials of ten repetitions were performed.

Processing: The identifiable peak in Y-axis acceleration (peak acceleration) that immediately followed the beginning of the movement in the outward direction was taken as the dependent variable (measured in G's, the output unit of the app). The average of the ten trials was taken as the outcome for each measure. Slower arm speeds (smaller peak acceleration values) were taken to indicate worse motor performance.

##### Leg Withdrawal

The purpose of this task was to create a standardized, iPod-measurable outcome that would simulate the ability to ballistically withdraw the leg in an upward direction as is required in rapid transition from the accelerator to brake pedal during driving. The focus was on the upward phase of the movement.

Setup: An iPod was firmly attached to the distal side of the participants lower leg (above the ankle) while in a seated position.

Directions: Instructions were as follows: “*Every time you feel a tap on the iPod, lift your leg and foot vertically about 6 inches as fast as possible, and then return your heel to the ground, keeping your ankle flexed and toes off the ground*.” During this task participants kept their eyes closed, their hands clasped together at the waist, not resting on the legs. Two trials of ten repetitions were performed (each with a pseudorandom interval of 2–5 s after the leg came to rest).

Processing: As with the arm movement task, the peak in Y-axis acceleration (peak acceleration) that immediately followed the beginning of the leg movement was taken as the dependent variable (measured in G's) with an average calculated from ten trials. Slower leg speeds (smaller peak acceleration values) were taken to indicate worse motor performance.

##### Index Finger Tapping

The goal of this task was to assess finger tapping speed, a validated measure of general motor function that has been used to assess fine motor control and simple motor speed after intoxication (42–46).

Setup: Participants were seated in a chair with their dominant forearm and palm resting on the corner of the iPod placed on a table.

Directions: The participants were instructed to: “*Tap the corner of the iPod with your index finger forcefully and consistently, as fast as possible, for 20 s, making sure to keep your hand flat while tapping*.”

Processing: The average number of taps per second (tapping rate) was calculated as the dependent variable. Slower tapping speeds (smaller tapping rate values) were taken to indicate worse general motor performance.

##### Postural Sway (Balance)

This task assesses changes in sway across three conditions, eyes open (EO), eyes closed (EC), and head tilted backwards with eyes closed (HBEC), as was described previously to assess proprioception and intoxication (12).

Setup: A Velcro-compatible elastic belt was wrapped tightly around the hips with an iPod firmly attached to the belt. Across trials and timepoints, the feet were placed 10% of body height apart. The hands were crossed in front of the chest.

Directions: Participants were directed to “*Stand as still as possible for 30 s with your eyes open, followed by 30 s with your eyes closed, followed by a final 30 s with your eyes closed and your head tilted slightly backwards, about 45*°.”

Processing: The order of conditions was the same for all subjects and time points. For each separate condition (EO, EC, HBEC) the standard deviation of acceleration (SD of Acceleration) was calculated as the dependent variable for the last 25s of each 30s trial. Greater SD of Acceleration values per condition were taken to indicate greater postural fluctuations (worsened balance). Methodological details can be found in Bidwell et al. (12).

### Primary Statistical Analysis

All statistical analysis was completed using SPSS (IBM Statistics v. 26). Motor performance was first assessed for systematic differences between the two trials at each timepoint, using a General Linear Model Repeated Measures Analysis of Variance (RMANOVA). In the absence of a significant Trial effect, the average values of the two trials were used as the dependent variable at each of the three timepoints. If the Trial effect was significant, the best value of two trials was used as the dependent variable (see *Task Trial Analysis* in Supplementary Material).

For each dependent variable, significant main and interaction effects of Time (Pre-Use, Acute Post-Use, 1 h Post-Use) and Sex (Female, Male) are reported. *A priori* contrasts were employed based on the design and goals of the study. The contrasts assessed the significance of changes between timepoints and interactions between independent variables (e.g., sex) and time. Therefore, there was no correction of the *P* < 0.05 significance level within each family of comparisons (e.g., arm, leg, index finger, and balance tasks). The change in cannabinoid levels over the three concentrate use timepoints are reported elsewhere (12).

#### Demographics and Cannabis Use

Prior to the main analyses, female vs. male concentrate users were compared on relevant demographic characteristics. To test sex differences on race a χ^2^-test was used, while *t*-tests were utilized to test sex differences in continuous measures (age, body mass index, and cannabis use measures).

#### Motor Performance Effects

For the arm, leg, index finger, and whole-body balance tasks a separate RMANOVA, one per task, was used to assess changes in motor performance after concentrate use and whether changes in performance across time were different between men and women. Extending previous balance findings (12), we completed a priori contrasts for each balance Condition (Eyes open, Eyes closed, Head back eyes closed) by Sex. The within-participant independent variable of Time and the between-participants independent variable of Sex were tested as main effects and the Time X Sex interaction was also tested.

#### Motor Performance and Cannabinoid Correlations

To determine whether a cannabis-related change in performance on one motor task was related to a cannabis-related change on another task, change scores between cannabis timepoints were computed for each significant motor outcome [(Acute Post-Use)–(Pre-Use), (1 h Post-Use)–(Pre-Use)]. Pearson correlations between the change in task performance acutely or 1 h after cannabis use was determined. Only tasks that demonstrated a significant change over time on performance in the primary repeated measure models were tested for associations.

Pearson correlations were also used to determine the relation between an acute change in motor task performance and an acute change in cannabinoid levels immediately after concentrate use. The acute change [(Acute Post-Use)–(Pre-Use)] in motor performance and the acute change in THC or 11-OH-THC levels [(Acute Post-Use)–(Pre-Use) were utilized in these analyses.

## Results

### Sample Characteristics

Participant (*N* = 65) characteristics are summarized in Table 1. Males reported initiating cannabis use at an earlier age and spent less time inside their home between the mobile Pre-Use and Acute Post-Use timepoints compared to females. Other demographic and cannabis use measures were not significantly different by sex.

**Table 1:** Demographic and cannabis use history by sex.

**Measure**	**Total overall**	**Sex group**	
		**Female**	**Male**
**Demographics**			
*N* (% of total)	65	30 (47%)	35 (53%)
Age (years)	27.88 ± 9.49	26.63 ± 9.08	28.94 ± 9.83
Race (% White)	69%	73%	66%
Body mass index (kg/m^2^)	24.13 ± 3.82	23.65 ± 4.66	24.54 ± 2.92
**Cannabis use (Baseline)**			
Regular Cannabis Use Onset (age in years)	17.13 ± 2.86	18.02 ± 3.15	^*^16.35 ± 2.36
^a^Marijuana dependence (0–11)	3.17 ± 2.20	3.37 ± 2.30	3.00 ± 2.13
^b^Overall cannabis use (days/month)	25.83 ± 5.33	25.50 ± 4.91	26.11 ± 5.72
^b^Concentrate use (days/month)	17.02 ± 11.04	15.37 ± 9.55	18.43 ± 12.12
^b^Dabs of concentrate (times/day)	5.13 ± 5.15	4.24 ± 3.70	5.96 ± 6.16
^b^Flower use (days/month)	14.94 ± 10.77	16.90 ± 9.66	13.26 ± 11.52
^b^Drags of flower (times/day)	10.84 ± 7.90	9.91 ± 7.35	11.71 ± 8.41
**Cannabis use (acute post-use)**			
^c^Concentrate amount used (grams)	0.13 ± 0.19	0.15 ± 0.22	0.12 ± 0.15
Time out of van/spent dabbing (min)	13.18 ± 6.19	15.23 ± 7.17	^*^11.40 ± 4.61

*Participant [N (% of total sample)] demographics and the average (mean ± SD) value is reported for each measure (units)*.

a *Marijuana Dependence Composite Score*.

b *Timeline Follow-Back (30-day)*.

c *Amount of study cannabis participant weighed by scale in their home and self-administered during the mobile appointment. Similar Total Overall data previously reported (12)*.

* Significant difference (t-test, ^*^p < 0.05) by sex (male vs. female) denoted. Comparisons were conducted separately for each outcome measure.

### Motor Performance After Concentrate Use

Table 2 reports the mean % change in motor performance and the repeated measure and within-participant *post-hoc* contrast results between cannabis concentrate timepoints.

**Table 2:** Effects of concentrate use over time on motor performance.

**Measure**	**^a^Main effect of Time**	**Stat**	**^b^Pairwise effects by timepoint**			**Summary**
			**Pre vs. Acute**	**Acute vs 1 h**	**Pre vs. 1 h**	
**Arm speed**	*F*_(1.692, 103.197)_ = 26.605, *p* < 0.001	% Δ:	−15	−1	−16	Acute & 1 h impairment
		*p:*	^*^0.000	0.52	^*^0.000	
**Leg speed**	*F*_(1.782, 108.724)_ = 3.238, *p* = 0.049	% Δ:	−2	−6	−7	1 h impairment
		*p:*	0.58	^*^0.026	^*^0.033	
**Tap speed**	*F*_(2, 122)_ = 2.350, *p* = 0.100	No main effect of time				
**Postural sway**						
Eyes Open	*F*_(2, 124)_ = 3.411,	% Δ:	14	−4	8	Acute impairment
	*p* = 0.036	*p:*	^*^0.017	0.32	0.11	
Eyes Closed	*F*_(1.74, 107.64)_ = 4.227,	% Δ:	11	−7	3	Acute impairment
	*p* = 0.022	*p:*	^*^0.013	0.062	0.36	
Head Back/Eyes Closed	*F*_(2, 124)_ = 0.053, *p* = 0.95	No main effect of time				

a Repeated measure main effect of Time and

b Pairwise differences reported between timepoints: before (Pre-Use) and after (Acute Post-Use and 1 h Post-Use) cannabis concentrate use, by mean % change (% Δ) and p-value (^*^p < 0.05).

#### Arm Extension Task

**Figure 2:**
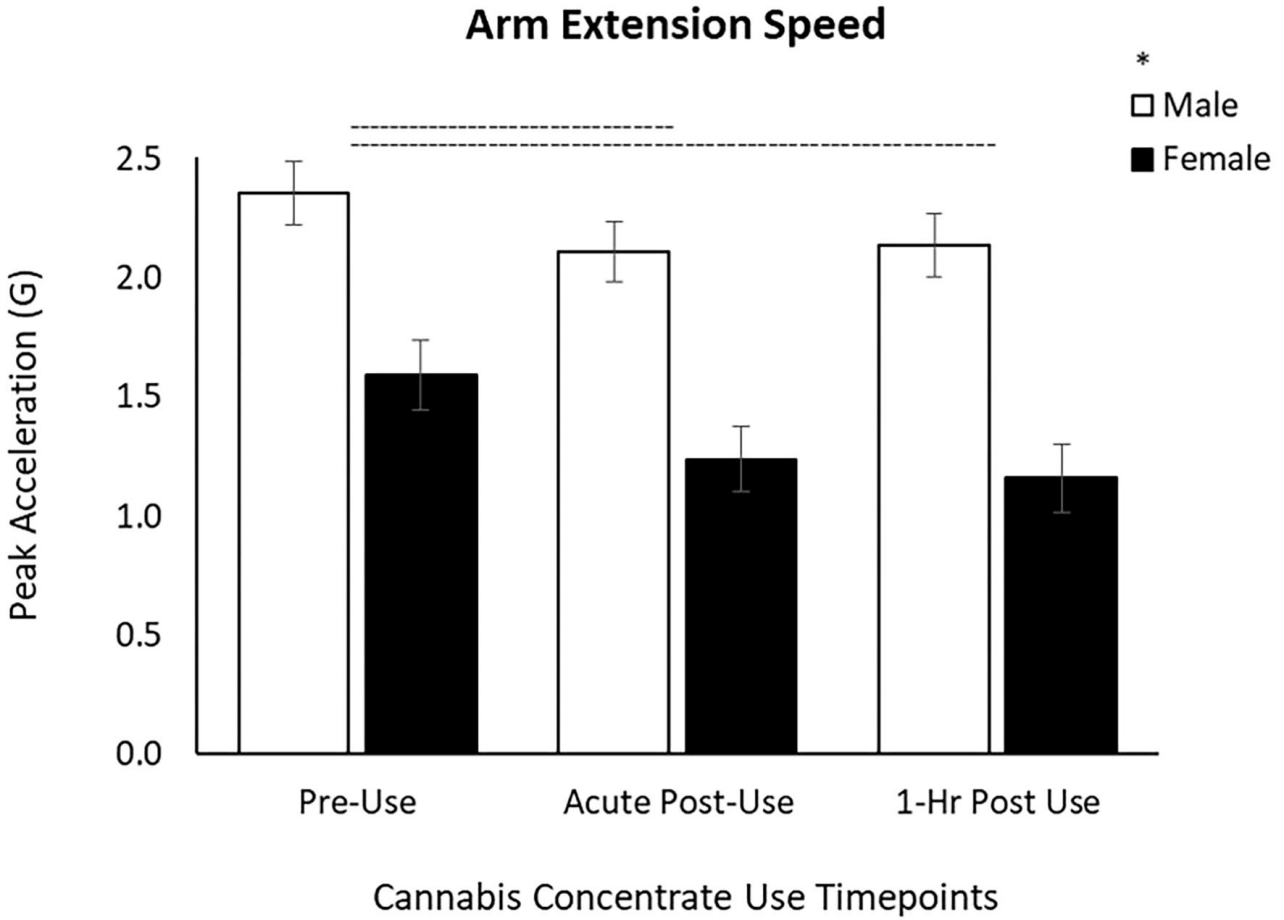
Arm extension speed decreases by 15% after concentrate use from Pre-Use to Acute Post-Use and by 16% and from Pre-Use to 1 h Post-Use in male and female users. Male arm extension speed is greater than females, yet the response to cannabis concentrate is similar between sexes (no Time x Sex interaction). (–) Main Time effect followed by pairwise comparisons denoted between timepoints; (^*^) Main Sex effect denoted above key (*p* < 0.05).

For the arm task, there was a main effect of Time [*F*_(1.69, 103.12)_ = 26.6, *p* < 0.001] on arm speed and a main effect of Sex [*F*_(1, 61)_ = 22.2, *p* < 0.001]. *Post-hoc* pairwise comparisons showed that arm speed was slowed by 15% from Pre-Use to Acute Post-Use (*p* < 0.001) and by 16% from Pre-Use to 1 h Post-Use (*p* < 0.001) (Table 2, Figure 2). There was no difference between Acute and 1 h Post-Use timepoints (*p* = 0.52). Men extended their arm faster than women, however the changes over time were not different between sexes (Time x Sex *p* = 0.097; Figure 2).

#### Leg Withdrawal Task

**Figure 3:**
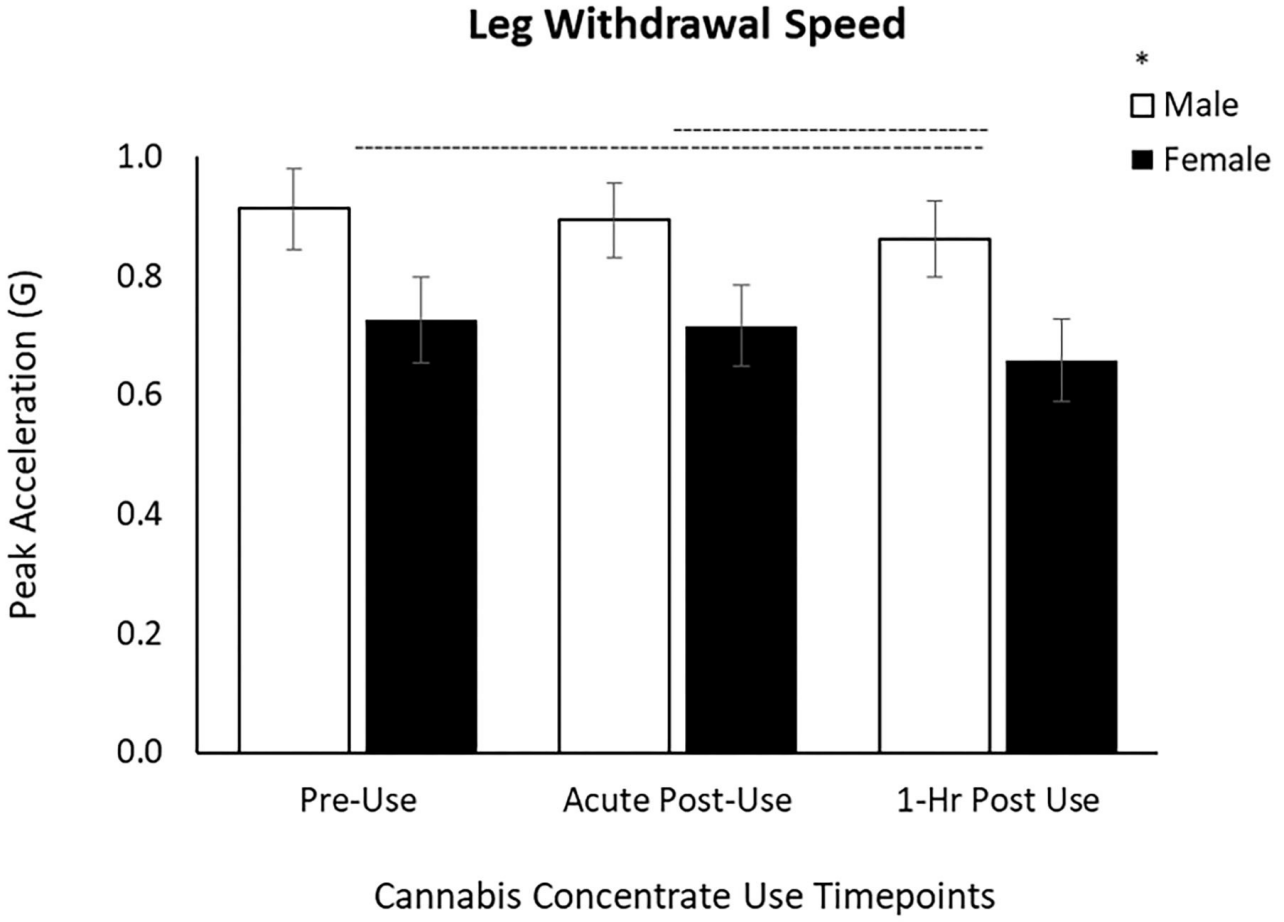
Leg withdrawal speed decreased by 6% between Acute and 1 h Post cannabis concentrate use and by 7% from Pre-Use to 1 h Post-Use in male and female users. Male leg withdrawal speed is greater than females, yet the response to cannabis concentrate is similar between sexes (no Time x Sex interaction). (–) Main Time effect followed by pairwise comparisons denoted between timepoints; (^*^) Main Sex effect denoted above key (*p* < 0.05).

For the leg task, there was a main effect of Time [*F*_(1.78, 109)_ = 3.24, *p* = 0.049] and Sex [*F*_(1, 61)_ = 4.33, *p* = 0.042]. *Post-hoc* pairwise comparisons demonstrated a significant slowing from the Pre-Use timepoint to 1 h Post-Use (*p* = 0.033) and between the Acute and 1 h Post-Use (*p* = 0.026) timepoints (Table 2, Figure 3) with no difference between the Pre-Use and Acute-Post-Use timepoint (*p* = 0.58). As with arm speed, men moved the leg faster than women but there was no Time x Sex interaction (*p* = 0.86; Figure 3).

#### Index Finger Tapping Task

There was no main effect of Time (*p* = 0.10, Table 2) but a main effect of Sex [*F*_(1, 61)_ = 5.79, *p* = 0.019] on index finger tapping rate. Index finger tapping was significantly faster for men than women, but the responses over time were not different between sex (Time x Sex interaction *p* = 0.64).

#### Postural Sway Balance Tasks

These results extend our previous findings of a significant decrease in postural stability, across increasingly difficult balance tasks (Condition: EO, EC, and HBEC) as well as a significant quadratic effect of Time found only for the EC condition in a sample of flower and concentrate users. To determine: (1) overall balance differences between Sex and (2) differences across Time based on Sex from only the sample of concentrate users we assessed each balance condition (EO, EC, and HBEC) over Time (between individual timepoints) and by Sex.

##### EO Balance

There was a main effect of Time [*F*_(2, 124)_ = 3.41, *p* = 0.036, Table 2], and neither a main effect of Sex (*p* = 0.88), nor an interaction of Time x Sex (*p* = 0.52). After using cannabis concentrate, EO postural sway increased at the Acute Post-Use timepoint (*p* = 0.017) but 1 h Post-Use did not differ from Pre-Use (*p* = 0.11) or Acute Post-Use (*p* = 0.32; Table 2).

##### EC Balance

There was also a main effect of Time [*F*_(1.74, 107.64)_ = 4.23, *p* = 0.022], with postural sway increasing acutely from Pre-Use to Acute Post-Use (*p* = 0.013) with no difference between Pre-Use and 1 h Post-Use (*p* = 0.36) or between Acute- and 1 h Post-Use (*p* = 0.062; Table 2). Like EO, there was neither a main effect of Sex (*p* = 0.88) nor an interaction of Time x Sex (*p* = 0.99).

##### HBEC Balance

There was no main effect of Time (*p* = 0.95, Table 2), Sex (*p* = 0.85), or Time x Sex (*p* = 0.33).

#### Motor and Cannabinoid Correlations After Concentrate Use

##### Motor × Motor Correlations

To determine whether a change in performance on one motor tasks was related to a change in another motor task after concentrate use, change scores were computed between Pre and Acute and between Pre and 1 h timepoints, for each motor outcome. The change in performance from Pre-Use to Acute Post-Use was positively correlated between EO and EC postural sway (*r*_64_ = 0.381, *p* = 0.002), and between arm and leg speed (*r*_63_ = 0.348, *p* = 0.005). However, the change in performance from Pre-Use to Acute Post-Use was not associated between arm speed and EO sway (*p* = 0.88) or between arm speed and EC sway (*p* = 0.70). A modest positive association was shown between the arm speed and leg speed change scores from Pre-Use to 1 h Post-Use (*r*_63_ = 0.289, *p* = 0.022) but no other significant between-task correlations were found.

##### Motor × Plasma Correlations

For the motor tasks that changed significantly after acute concentrate use (arm speed, EO balance, and EC balance), we determined whether this change was correlated to acute changes (12) in plasma cannabinoid levels. Change scores were computed between Pre-Use and Acute-Use for each motor and cannabinoid outcome. To determine if a significant acute change in performance on motor tasks (arm, EO, and EC) is related to an acute change in THC-related plasma levels (plasma THC or 11-OH-THC) after concentrate use, change scores were computed between Pre-Use to Acute Post-Use timepoints for those three motor and two plasma outcomes. In total, there were only two weak positive associations, between the Pre-Use to Acute Post-Use change in EO postural sway and the change in plasma levels of THC (*r*_64_ = 0.247, *p* = 0.049) and 11-OH-THC (*r*_64_ = 0.296, *p* = 0.017).

## Discussion

This report on basic motor impairment after the acute use of cannabis concentrates shows altered performance on a battery of motor tasks in frequent users. Cannabis concentrate decreased limb speed with arm and leg peak acceleration slowing 1 h after use (16 and 7%, respectively). Although men were faster overall for the motor speed tasks, cannabis-induced impairment was not different between women and men. Balance was acutely impaired after concentrate use, both with eyes open and closed (by 14 and 11%, respectively), yet there was no difference in impairment between men and women. In general, cannabis concentrate-induced motor impairments were correlated between arm and leg speed tasks and between balance conditions. However, the rise in acute post-use plasma THC levels (12) was not correlated with acute impairments of speed or balance. The results can inform researchers about future investigational targets on basic motor performance and allow more precise risk assessments to be made by policy makers regarding the impact of cannabis concentrate use on motor impairment.

### Arm and Leg Speed Are Impaired After Cannabis Concentrate Use in Frequent Users

This is the first study to investigate movement speed after naturalistic use of cannabis concentrates. The tasks were simple in that they measured the pure ability to generate a rapid, discrete, large-amplitude descending motor command to accelerate an unloaded limb rapidly–with little contribution from sophisticated cognitive processing. There is little previous research assessing cannabis intoxication with simpler motor tasks. Despite no directly comparable findings in the literature, these results can be contextualized by comparing our conclusions with prior work in more complex psychomotor tasks after low-potency cannabis use. Two reports were conducted in small samples of users who were administered low-potency THC in a lab setting and used complex tasks that required a combination of reaction time, cognitive demand, and gross motor speed. The most comparable previous measure to our arm extension task was a target reaching task in response to a choice visual stimulus (47). In that study there was no effect in response speed or accuracy 30-min after THC administration. In a driving simulator study, significant increases in steering variability, decreases in driving speed, and increases in choice reaction time suggests an acute cannabis-induced decrease in motor processing and complex motor speed (15). The present results indirectly expand this conflicting literature in complex arm-related tasks, by confirming an acute and 1 h cannabis concentrate impairment in simple ballistic arm speed.

In the lower limb, the results of Liguori et al. were conflicting in that there was no cannabis effect on braking latency but a decreased ability to maintain a set driving speed in driving simulations (27). Notably, this driving simulation was completed 20–30 min after smoking a low-potency flower cannabis cigarette (up to 3% THC). This begs the question of whether leg movement latency and driving speed (both requiring multiple domains) contain a contribution from raw leg speed impairment, and of whether the timing of impairment is different with concentrated THC products, in that our data shows stronger evidence of impairment at the 1 h timepoint. While past psychomotor and driving simulator studies were necessarily more complex and required multiple domains and movements to be tested simultaneously, our battery of tasks was focused on the production of simple movements isolated to one limb. The reporting of isolated arm and leg speed impairment provides new information on subtle domain-, movement-, and time-specific effects in frequent concentrate users.

### Balance With and Without Visual Feedback Is Acutely Impaired After Cannabis Concentrate Use

As with the acute impairments in arm speed, balance ability both with and without the benefit of visual feedback was acutely impaired after concentrate use but normalized after 1 h. In agreement, early research with low-potency cannabis (48) showed impaired balance (wobble board) that worsened as the dose of THC increased. Similarly, Hosko et al. (49) found decreased one-legged balance ability with eyes closed after administration of edible low-potency cannabis, consistent with our finding of impaired balance after high-potency cannabis use. Additionally, a study in experienced cannabis users also supports our findings with a general equilibrium score (as measured by body sway) increasing by ~11% after smoking the highest dose of flower cannabis tested (3% THC) (27). A cannabis cigarette with 3% THC is modest in potency compared with the typical concentrated product, yet the magnitude of effect was similar with 14 and 11% impairment found in our eyes open and closed tasks after concentrate use in frequent users. This suggests that tolerance to THC has increased with current market trends or that balance ability under these conditions has a ceiling of impairment. Future research needs to determine whether motor performance can be used as a consistent marker of cannabis impairment, especially as it becomes more evident that neither tolerance nor acute plasma THC levels can predict the extent of balance impairment.

Extending prior findings on balance ability (12), the current report has examined balance performance in relation to concentrate use specifically and in more detail. We consider potential sex effects, correlations with plasma THC levels, and the relationship of cannabis-induced changes in balance with changes in other features of motor performance. Postural sway increased acutely after concentrate use but recovered to Pre-Use levels by 1 h, with and without vision. This suggests responsiveness in the balance task and an effect of cannabis concentrates on the neural processing necessary for postural stability. Visual feedback is known to be a dominant source of sensory feedback during postural control. Accordingly, the availability of vision typically reduces postural fluctuations compared with eyes closed (50), suggesting that impairment was robust in concentrate users if detectable even with the benefit of visual feedback. This within-subject design and the relatively large number of participants made it possible to detect small but significant differences in balance after concentrate use in a brief, remotely deployable smart device-based motor battery. There was no effect of cannabis concentrate on head-back balance, a condition designed to disturb vestibular feedback and challenge balance control. This could be further explored with different types of users, cannabis administration paradigms, or increased task complexity, to provide more precise information on cannabis and proprioception-challenged balance.

### Motor Impairment Is Similar Between Men and Women

An overall difference in motor performance between sexes has been well-established, especially for ballistic speed (51, 52). The observed sex differences are therefore expected and indicate that such differences are detectable with a smart device-based, portable movement battery deployed in a mobile, vehicular lab setting. Notably, our large sample and nearly equal number of males and females is a departure from most existing cannabis literature [e.g., (53–55)] and is a strength of this report focused on cannabis and sex effects. To report that cannabis concentrate alters balance, arm speed, and leg speed similarly between males and females, despite documented sex differences in general cannabis use patterns and effects (33, 56–59) fills a critical gap in the cannabis literature (16, 60). The similarity of cannabis effects between males and females may allow for more effective application of impairment testing in future prevention and policy efforts as cannabis use prevalence has begun to converge between women and men (61).

### Motor Impairment Is Largely Uncorrelated With Plasma THC Levels

A lack of correlation between plasma cannabinoid levels (THC and 11-OH-THC) and psychomotor effects is in line with most of the cannabis intoxication literature to date (18, 21, 26). For example, Boggs et al. (47) demonstrated that increases in THC plasma levels (5-min after smoking low-potency THC cannabis) were not correlated with either impairment in complex upper and lower limb psychomotor measures, or with subjective intoxication. This agrees with our findings. However, the ability of the present dataset to provide information on potential correlations between impairment in domain-specific basic motor performance (limb speed, whole-body balance, finger movement) at quite high blood cannabinoid levels is largely novel and represents a substantive addition to the cannabis field.

With only a minor correlation found between the change in eyes open balance and cannabinoid levels, no potential effect of sex on balance, and no correlation between the acute change in arm or leg speed and the acute change in cannabinoids, the data suggests that blood cannabinoid levels do not predict the severity of acute physical impairment, at least on these tasks. This means that plasma THC level is limited in precision to predict functionally relevant movement impairment. Although this idea remains under-investigated, with little comparable research on basic motor performance after concentrate use, these findings at least suggest that plasma cannabinoid levels may not be the best measure of physical impairment. This also suggests that public policy needs to be better informed by basic, observational, clinical, and potentially industry research (to better access current market products that are federally regulated). Lastly, this highlights the need to remain critical of our common sobriety measures and to be open to novel investigational methods and devices.

### Limitations and Significance for Cannabis Policy

To exclude a potential contribution of time related factors (e.g., boredom, fatigue, learning/testing effects) other than acute cannabis effects, it would be optimal to compare all results in the cannabis-use participants to a non-concentrate use control session in the same participants or to a non-concentrate use control group. We considered the possibility that the time between trials and timepoints could alter performance in a similar manner (fatigue within or between cannabis timepoints) and thus we reported any trial by time effects on performance in the supplemental report and calculated our dependent outcomes accordingly. However, if the time between timepoints (~60 min) contributed largely to effects, one might expect all tasks would have a similar pattern of impairment over time, which was not the case. This does not entirely rule out these or other potential contributors to the performance declines but does lend support for acute cannabis being a primary contributor to impairments.

These movement speed and balance impairments reported in highly experienced users indirectly support survey-based association studies that positively linked frequency of cannabis use and THC with injuries (i.e., culpability of road traffic accidents, injuries inside and outside of work, minor injuries/accidents, etc.) (62–64). Since recreational cannabis and cannabis use research is legal only for those 21 or older, our results cannot be directly translated to those younger than 21.

The generalizability of balance results to daily living, is high in the sense that adequate control of the body's upright stance is critical for function and safety in humans. Postural stability (balance) is a common component of sobriety assessment and is accepted as a generalized measure of motor control. The ballistic arm and leg measures and finger tapping task were designed to assess raw movement speed, as opposed to the ability to perform a complete functional movement or series of movements as might be required in activities of daily living, driving, and work. A limitation of this approach is that our measure of standardized, abbreviated movement of the isolated limb is only part of a more complex movement that would be required in real life (brake pedal operation, reactive steering during driving, operating machinery etc.). The advantage is the ability to capture precise measures of speed and motor control that contribute to more complex movements in daily living, all using a smartphone-based app in a community-based sample. We believe these to be the first mobile assessments of motor performance in the context of cannabis intoxication.

Methodologically, this report tested within-subject effects before and after using high potency THC in frequent users. These results may therefore not reflect effects that might be observed in novice cannabis users. It is also possible that a much larger sample overall could increase the power to detect effects that in the present data are either borderline significant (i.e., a decrease in tapping rate over time) or non-existent (i.e., an interaction between sex and cannabis use over time). This experimentally derived report balances internal and external validity, using a within subjects design and *ad-libitum* administration of dispensary-grade cannabis concentrate to test effects of high potency cannabis on motor outcomes. The findings may be particularly useful in states that see an increase in the number of frequent concentrate users after legalizing recreational cannabis (65). The results should aid assessment of occupational risk, longitudinal and between-user public health study design, and data-driven policy.

## Conclusions

These findings demonstrate the feasibility of a multi-task, mobile motor performance battery and the utility of combining this with acute measures of plasma cannabinoid levels after naturalistic cannabis administration. The increasingly popular use of concentrated cannabis impairs some, but not all features of motor performance. These findings provide the first evidence that concentrated cannabis slows arm and leg speed. This confirms the importance of assessing basic features of motor performance (i.e., without cognitive demands) after concentrate use. The results also demonstrate that changes in plasma cannabinoid levels are not correlated with limb speed impairments and only weakly correlated with the degree of balance impairment. Additionally, the cannabis concentrate effect on limb speed and balance is not different between men and women. Notably, motor effects are largely without meaningful correlation with plasma cannabinoid levels, highlighting a critical issue in past and future research, clinical evaluations, professional/work settings, legal assessment of cannabis intoxication, and public health and policy.

## Data Availability Statement

The raw data supporting the conclusions of this article will be made available by the authors, without undue reservation.

## Ethics Statement

The studies involving human participants were reviewed and approved by University of Colorado Boulder Institutional Review Board. The patients/participants provided their written informed consent to participate in this study.

## Author Contributions

LH: data curation, formal analysis, investigation, methodology, project administration, resources, software, validation, visualization, writing–original draft, and writing–review and editing. BT: conceptualization, formal analysis, methodology, project administration, resources, software, supervision, validation, writing–original draft, and writing–review and editing. AB and KH: conceptualization, funding acquisition, methodology, and writing–review and editing. LB: conceptualization, funding acquisition, methodology, project administration, resources, software, supervision, and writing–review and editing. All authors contributed to the article and approved the submitted version.

## Conflict of Interest

The authors declare that the research was conducted in the absence of any commercial or financial relationships that could be construed as a potential conflict of interest.
