# Supplementary material for: Acute Effects of Cannabis Concentrate on Motor Control and Speed: Smartphone-Based Mobile Assessment
Source: Front Psychiatry. 2021 Jan 22;11:623672. doi: 10.3389/fpsyt.2020.623672 (PMC7862106; doi:10.3389/fpsyt.2020.623672)
Supplement: Supplementary file 1 [file Data_Sheet_1.pdf]

Supplementary Material

1 Supplemental Methods Data

1.1 Materials, Setup & Data Processing

The back of the iPod Touch that was used to sense movement was covered in adhesive Velcro. A Velcro-compatible elastic band was used to firmly secure the device to the subject. In all tasks except Finger Tapping, the iPod was further secured with two smaller strips of Velcro (0.25 x 15 x 1.3 cm). In all tasks except Balance, participants were seated in a chair (seat width: 38 cm, seat depth: 22 cm, seat height: 44.5cm, back height: 93cm).

The iPod device was lightly tapped before and after each Trial to introduce a clear mechanical artifact into the signal and ensure identification of the beginning and end of each Trial during the subsequent offline analysis. The data logging App was used to record triaxial acceleration and rotation signals (X, Y, and Z axes) from the iPod Touch's sensors at a 100Hz sampling rate. The data were saved in a text file on the device. All data was manually visually inspected before subsequent offline analysis.

1.2 Motor Function Tasks

1.2.1 <u>Arm Extension</u>

Setup: Participants were seated in a chair in the mobile laboratory with the elbow of the dominant arm at a right angle, the forearm semi-prone and parallel to the floor, and upper arm slightly abducted away from the trunk. A wide Velcro strap was firmly wrapped around the distal forearm above the wrist. The iPod Touch was firmly attached to the strap on top of the forearm (screen face up) and further secured with two small Velcro straps. The end of the iPod was aligned with the styloid process of the radius. The device was attached so that the Y-axis of the device was aligned with the direction of forearm movement in the sagittal plane. An investigator was positioned aside the participant to tap the iPod with a reflex hammer to provide a tactile stimulus for the participant. The hammer was out of view of the participant so that they were unaware of the timing of the tap.

Processing: The tap stimulus produced a sharp and readily identifiable deflection in the acceleration signal, followed by the identifiable start of the movement in the outward direction (Y-axis acceleration).

1.2.2 <u>Leg Withdrawal</u>

Setup: Participants were seated in a chair with the back straight, hip and knee joints at a right angle, and feet flat on the ground. An elastic band was wrapped around the lower leg just above the ankle. The iPod was attached to the strap on the lateral aspect just above the lateral malleolus (home button toward the ankle). The iPod was further secured with two small Velcro straps.

Processing: As with the arm movement task, the hammer tap produced a sharp peak in acceleration and the subsequent upward movement of the leg was readily identifiable in the Y-axis acceleration signal. The Y-axis peak acceleration during the upward movement of the leg was measured after each hammer tap. The continuous dorsiflexion requirement ensured that the participant did not propel the leg upward with active contractions of the plantarflexors.

1.2.3 Index Finger Tap

Setup: Handedness was determined via self-report. Participants were seated in a chair in the mobile lab with the dominant hand palm down and the forearm entirely on the table at a comfortable angle in front of their body. The iPod was placed on top of a 3mm-thick neoprene pad (14 x 7.6 cm) to prevent slippage on the desk and positioned so the lower right corner of the face of the device was under the tip of the index finger.

Processing: Each tap produced a clearly identifiable deflection in the pitch signal (gyroscope sensor) due to the small movement of the device from each tap. A combination of automated peak-detection algorithm and manual inspection (Spike 2 data analysis program) was used to determine the timestamp of the peak of each tap deflection.

1.3 Task Trial Analysis

For each task, a preliminary set of repeated measure ANOVAs were completed across Trial (Trial 1, Trial 2) and Time (Pre-Use, Acute Post-Use, One-Hour Post-Use) to assess potential between-Trial learning or practice effects in dependent motor outcomes: The dependent outcomes were Peak Acceleration for the arm and leg speed task, Tap Rate for the index finger, and SD of Acceleration for the three balance tasks (Eyes Open-EO, Eyes Closed-EC, Head Back Eyes Closed - HBEC). When there was no significant main effect of Trial and no interaction of Trial x Time ($p>0.05$), the average of Trials 1 and 2 was used as the dependent outcome at each timepoint. In the case of a significant Trial effect (main effect or Time x Trial interaction), the best value (rather than the average value) of two Trials was used as the dependent variable for that task.

1.3.1 Arm Extension (Peak Acceleration - Speed): A main effect of Trial was found for arm speed ($p=0.019$). Therefore, the best (fastest) values were used for the analysis of arm speed (higher peak acceleration values).

1.3.2 Leg Withdrawal (Peak Acceleration - Speed): There was no main effect of Trial ($p=0.31$) or an interaction of Trial x Time for leg speed ($p=0.44$). The average of Trials 1 and 2 was used as the dependent outcome for leg speed (higher peak acceleration values).

1.3.3 Index Finger Tapping (Tapping Rate - Speed): A main effect of Trial ($p<0.001$) on tapping rate was found, with faster performance on Trial 1 on average, but no interaction of Trial x Time ($p=0.30$). The best (fastest) Trial performance was used for the analysis of tapping speed (higher tapping speed values).

1.3.4 Postural Sway (SD of Acceleration - Balance): There was no main effect or interaction of Trial by Time (*p-values*>0.05) for the balance outcomes (EO, EC, HBEC), therefore the average of Trials 1 and 2 was used as the dependent outcome for whole body postural sway.
